# Clinical Features of Probable Cluster Headache: A Prospective, Cross-Sectional Multicenter Study

**DOI:** 10.3389/fneur.2018.00908

**Published:** 2018-10-26

**Authors:** Jong-Hee Sohn, Yun-Ju Choi, Byung-Kun Kim, Pil-Wook Chung, Mi Ji Lee, Min Kyung Chu, Jin-Young Ahn, Byung-Su Kim, Tae-Jin Song, Kyungmi Oh, Kwang-Soo Lee, Soo-Kyoung Kim, Kwang-Yeol Park, Jae Myun Chung, Heui-Soo Moon, Chin-Sang Chung, Soo-Jin Cho, Jeong-Wook Park

**Affiliations:** ^1^Department of Neurology, Chuncheon Sacred Heart Hospital, Chuncheon, South Korea; ^2^Department of Neurology, Presbyterian Medical Center, Jeonju, South Korea; ^3^Department of Neurology, Eulji Hospital, Seoul, South Korea; ^4^Department of Neurology, Kangbuk Samsung Hospital, Sungkyunkwan University School of Medicine, Seoul, South Korea; ^5^Department of Neurology, Samsung Medical Center, Seoul, South Korea; ^6^Department of Neurology, Severance Hospital, Seoul, South Korea; ^7^Department of Neurology, Seoul Medical Center, Seoul, South Korea; ^8^Department of Neurology, Daejin Medical Center, Bundang Jesaeng General Hospital, Seongnam, South Korea; ^9^Department of Neurology, Ewha Womans University School of Medicine, Seoul, South Korea; ^10^Department of Neurology, Korea University College of Medicine, Seoul, South Korea; ^11^Department of Neurology, Seoul St. Mary's Hospital, Catholic University of Korea College of Medicine, Seoul, South Korea; ^12^Department of Neurology, Gyeongsang National University College of Medicine, Jinju, South Korea; ^13^Department of Neurology, Chung-Ang University Hospital, Seoul, South Korea; ^14^Department of Neurology, Inje University College of Medicine, Seoul, South Korea; ^15^Department of Neurology, Dongtan Sacred Heart Hospital, Hwaseong, South Korea; ^16^Department of Neurology, Uijeongbu St.Mary's Hospital, Uijeongbu, South Korea

**Keywords:** cluster headache, autonomic symptom, probable diagnosis, definite diagnosis, trigeminal autonomic cephalalgia

## Abstract

**Background:** Epidemiological data of probable cluster headaches (CH) are scarce in the relevant literature. Here, we sought to assess the prevalence and clinical characteristics of probable CH in comparison with definite CH.

**Methods:** Data used in this study were obtained from the Korean Cluster Headache Registry (KCHR), a prospective, cross-sectional, multicenter headache registry that collected data from consecutive patients diagnosed with CH.

**Results:** In total, 159 patients were enrolled in this study; 20 (12.6%) were diagnosed with probable CH. The most common unfulfilled criterion in patients with probable CH was the duration of attack, which was found in 40% of patients with probable CH. Among clinical characteristics, the number of autonomic symptoms tended to be lower in probable CH than in definite CH (1.7 ± 1.2 vs. 2.4 ± 1.5, *p* = 0.051) and conjunctival injection and lacrimation showed an increased odds ratio (OR) [OR = 3.03; 95% confidence interval (CI): 1.03–8.33] in definite CH. The groups did not differ with regard to baseline demographic characteristics, disability, impact on life, or treatment response.

**Conclusions:** Probable CH is relatively common among CH disorders, with a clinical impact similar to that of definite CH.

## Introduction

Cluster headache (CH), which is characterized by severe headache localized in or around the eye and accompanied by cranial autonomic symptoms (CAS), is the most painful form of primary headache disorder. Based on epidemiological surveys from the USA and Europe, CH has a prevalence of 0.1% in the general population (1). The diagnosis of CH is determined based on the presence of a strictly unilateral headache attack lasting 15–180 min and occurring up to eight times a day. Headaches must be accompanied by at least one autonomic symptom ipsilateral to the pain, a sense of agitation, or both (2, 3). Episodic CH is the most common presentation, reported in 85–90% of patients with CH, with headaches occurring as a series of daily attacks lasting for weeks or months, followed by complete remission (4).

Probable CH is diagnosed when a patient's headache fulfills all but one criterion for CH based on the International Classification of Headache Disorder (ICHD) (2, 3). Diagnosis of this group of headache sufferers is an important issue in clinical practice. Considering the similarity in the clinical characteristics and treatment response profiles, cases of probable CH may be driven by the same pathophysiological processes that drive definite CH. Despite this uncertainty in clinical evaluation, the incorporation of a probable diagnosis in headache classification may increase the likelihood that a patient will receive an accurate diagnosis and adequate treatment at their initial visit (5). If probable CH were found to be a prevalent form of CH, studies that focused entirely on definite diagnoses may have underestimated the significance and impact of probable CH. Probable migraine has been reported to be a common primary headache disorder with a prevalence of 4.5–14.6% in Western and Asian countries (5–11). However, epidemiological data assessing probable diagnoses of CH or trigeminal autonomic cephalalgia (TAC) remain limited. Previously, a study found the prevalence of suspected CH in 32 of 1,145 (2.7%) cases included in a community-based survey, whereas the prevalence of probable TAC among all TAC cases was 40.9% in a multicenter study of first-visit headache outpatients at neurology clinics (12, 13). Furthermore, neither the clinical characteristics of probable CH nor its effects on the quality of life of sufferers have been reported.

Here, we sought to assess the proportion of probable CH diagnoses and to clarify the diagnostic profile of individuals with probable CH. In addition, the clinical characteristics of probable CH patients were compared with those of patients diagnosed with definite CH.

## Methods

### Study design and patients

This study was performed using data obtained from the Korean Cluster Headache Registry (KCHR) study, a multicenter, cross-sectional headache registry that used prospectively collected data from consecutive patients with CH treated at the neurology outpatient departments of 15 hospitals in Korea between August 2016 and May 2018. This study was conducted at 13 university hospitals (8 tertiary referral hospitals and 5 secondary referral hospitals) and 2 secondary referral general hospitals throughout Korea in accordance with the Declaration of Helsinki and good clinical practice. Board-certified neurologists with a special interest in headache conducted the study, and all investigators were Directors on the Korean Headache Society Board.

All participants were examined by each investigator to confirm that the diagnosis fulfilled the criteria set forth in the International Classification of Headache Disorders, 3rd Edition, beta version (ICHD-3β) (2) for CH and asked to complete questionnaires in relation to the visit. Participants who met the diagnostic criteria for probable or definite CH based on ICHD-3β were identified at the initial interview, and their diagnoses were finally confirmed via a re-evaluation process at least 2 weeks later. The study protocol and informed consent form were reviewed and approved by the Institutional Review Board of each hospital. Written informed consent was obtained from all participants before their enrolment in the study.

### Clinical information and questionnaire

Investigators assessed the demographic features of and recorded the clinical information about each patient's current and previous bouts of CH. Clinical information regarding the current headache episode included location, severity, duration, and frequency of pain, associated symptoms, and duration of bout period. Previous history of CH included the time since the first bout of CH, the total frequency of the cluster period, and the pattern of recurrence.

Each patient completed a self-administered Headache Impact Test-6 (HIT-6) questionnaire to measure headache-related impact, the Patient Health Questionnaire-9 (PHQ-9) to assess depression, the Generalized Anxiety Dirorder-7 (GAD-7) to assess anxiety, the EQ-5D to measure of health-related quality of life, and the Short Form Perceived Stress Scale-4 (PSS-4) to assess psychological stress. The impact of headaches on an individual's quality of life, as determined based on HIT-6 scores, was defined as mild (≤49), some (50–55), substantial (56–59), and severe (≥60) impact. Cut-off points for depression and anxiety were defined as PHQ-9 scores ≥8 and GAD-7 scores ≥6, respectively.

### Statistics

For continuous variables, the Kolmogorov–Smirnov test was used to assess the normality of the distribution. After normality was confirmed, chi-square and student's *t-*tests were used to compare the nominal and the continuous variables, respectively. When normality was not confirmed, continuous variables were analyzed by Mann–Whitney *U*-tests. We compared the odds ratios (ORs) and 95% confidence intervals (CIs) for the characteristics of definite CH with those of probable CH through univariate regression analyses. All analyses were performed using the Statistical Packages for the Social Sciences for Windows ver. 22.0 (IBM, Armonk, NY, USA); *P* < 0.05 were considered statistically significant.

## Results

### Prevalence and diagnostic profiles of probable CH

**Figure 1:**
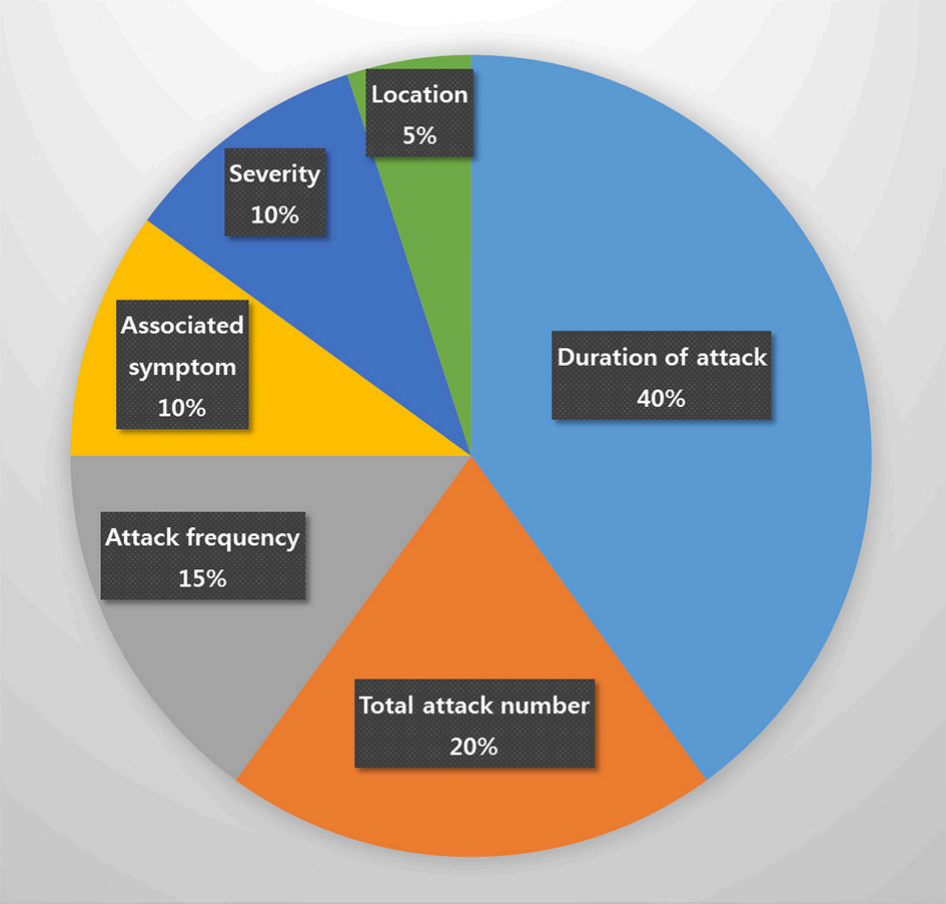
The reason for failure of definite cluster headache diagnosis.

In total, 159 patients, 20 (12.6%) of whom were diagnosed with probable CH, 114 (71.7%) with episodic CH, 5 (3.1%) with chronic CH, and 20 (12.6%) with unclassified CH, were enrolled in this study. The most commonly unfulfilled criterion in patients with probable CH was the duration of attack, which was found in eight (40%) patients with probable CH. The duration of the attack of probable CH (163.0 ± 164 min) tended to be longer than that of definite CH (94.3 ± 52.9 min), but the difference was not significant (*p* = 0.078). Specifically, it exceeded 180 min in six of eight patients and was under 15 min in two. Other unmet criteria necessary for a definitive diagnosis of CH included total attack number (4; 20%), headache frequency (3; 15%), associated autonomic symptom (2; 10%), pain severity (3; 10%), and headache location (1; 5%; Figure 1).

### Clinical characteristics of patients with definite and probable CH

No significant differences in the clinical characteristics of patients with definite and probable CH were observed, including with regard to mean age, male to female ratio, body mass index (BMI), alcohol use, and smoking status. Among the headache characteristics, patients with probable CH showed a tendency toward fewer CAS compared to those with definite CH (1.7 ± 1.2 vs. 2.4 ± 1.5, *p* = 0.051; Table 1). Among all CAS, conjunctival injection and lacrimation had an increased OR among those with definite CH (OR = 3.03; 95% CI: 1.03–8.33; Table 2). The headache duration of those with probable CH was longer than that of those with definite CH; however, this difference failed to reach statistical significance (163.0 ± 164.0 vs. 94.3 ± 52.9, *p* = 0.078).

**Table 1:** Comparison of demographics and clinical features between definite and probable cluster headache.

	**Definite CH (*N* = 139)**	**Probable CH (*N* = 20)**	***p***
Age (years)	39.1 ± 10.7(19–76)	42.5 ± 12.7(22–80)	0.197
Male sex (%)	117 (84.2%)	17 (85.0%)	1.000
BMI (kg/m^2^)	24.0 ± 3.4(14–35)	24.7 ± 3.4(20–34)	0.343
Current smoker (%)	65 (44.8%)	7 (35.0%)	0.455
Alcohol drinking (%)	71 (51.1%)	8 (40.0%)	0.492
Age of onset (years)	29.1 ± 13.2(9–78)	30.9 ± 11.7(16–59)	0.549
Total period of cluster headache (years)	8.7 ± 8.4(0–48)	8.2 ± 7.9(0–24)	0.815
Duration of bout (weeks)	7.5 ± 10.3(1–57)	5.9 ± 4.7(1–22)	0.329
Total bout number	8.2 ± 12.2(1–100)	9.0 ± 11.5(1–50)	0.778
Presence of seasonal variation	62 (48.1%)	5 (26.3%)	0.126
Presence of diurnal variation	71 (52.2%)	8 (40.0%)	0.435
Number of autonomic symptom	2.4 ± 1.5(0–7)	1.7 ± 1.2(0–4)	0.051
Frequency of headache (day)	2.2 ± 1.9(0.1–10)	1.8 ± 1.3(0.3–5)	0.336
Duration of headache (min)	94.3 ± 52.9(12–270)	163.0 ± 164.0(10–600)	0.078
Intensity of headache (NRS)	9.1 ± 1.1(5–10)	8.6 ± 1.8(4–10)	0.223

*The parentheses indicate all range of values. CH, cluster headache*.

**Table 2:** Univariable odds ratios for definite and probable cluster headache as function of clinical characteristics.

		**Definite CH (*N* = 139)**	**Probable CH (*N* = 20)**	**Univariable OR**		
				***P***	**OR**	**(95% CI)**
Headache location	Orbital, supraorbital, temporal	107 (85.0%)	17 (85.0%)	0.423	1.69	0.53–7.58
	Unilaterality	136 (97.8%)	19 (95.0%)	0.461	2.39	0.11–19.14
Headache intensity	Severe intensity	128 (92.1%)	17 (85.0%)	0.304	2.05	0.43–7.40
Associated symptoms	Conjunctival injection/lacrimation	118 (84.9%)	13 (65.0%)	0.035	3.03	1.03–8.33
	Nasal congestion/rhinorrhea	79 (55.8%)	11 (55.0%)	0.877	1.08	0.41–2.77
	Eyelid edema	32 (23.0%)	4 (20.0%)	0.763	1.20	0.40–4.40
	Forehead and facial sweating	39 (28.1%)	1 (5.0%)	0.055	7.41	1.46–135.38
	Forehead and facial flushing	22 (15.8%)	2 (10.0%)	0.500	1.69	0.44–11.12
	Ear fullness	13 (9.4%)	1 (5.0%)	0.528	1.96	0.36–36.61
	Miosis/ptosis	29 (20.9%)	2 (10.0%)	0.264	2.37	0.63–15.46
	Restlessness/agitation	63 (45.3%)	7 (35.0%)	0.387	1.54	0.43–7.40

*CH, cluster headache; OR, odds ratios; CI, confidence interval*.

### Psychiatric comorbidity, impact on quality of life, and treatment response in patients with definite and probable CH

Next, we assessed psychosomatic comorbidities, including anxiety, depression, and stress using GAD-7, PHQ-9, and PSS-4 scores, respectively. No significant differences were evident between patients with definite and probable CH. Similarly, the headache-related disabilities, as determined based on HIT-6 and EQ-5D scores, of patients with probable and definite CH (Table 3) were also similar. Oxygen and triptan were the most commonly reported acute treatments for definite and probable CH with steroids the most commonly used preventive therapy. The treatment response to these drugs did not differ between groups (Table 4).

**Table 3:** Psychiatric comorbidity and headache-related disability of individuals with definite and probable cluster headache.

	**Definite CH (*N* = 139)**	**Probable CH (*N* = 20)**	***p***
GAD-7 scores	7.6 ± 5.5(0–21)	6.1 ± 5.6(0–21)	0.297
Presence of anxiety (%)	82 (61.2%)	7 (38.9%)	0.121
PHQ-9 scores	7.2 ± 6.0(0–27)	7.9 ± 7.9(0–25)	0.605
Presence of depression (%)	56 (41.8%)	7 (36.8%)	0.872
PSS-4 scores	6.6 ± 2.8(0–16)	6.0 ± 3.2(0–11)	0.385
EQ-5D scores	0.85 ± 0.14(0.54–0.95)	0.84 ± 0.14(0.55–0.95)	0.640
HIT-6 scores	68.1 ± 7.7(42–78)	63.9 ± 11.2(42–78)	0.117
Presence of severe impact	112 (83.6%)	13 (65.0%)	0.094
ASC-12 scores	2.5 ± 3.9(0–20)	2.9 ± 4.1(0–16)	0.670
Presence of cutaneous allodynia	40 (33.6%)	8 (42.1%)	0.644

*The parentheses indicate all range of values. CH, cluster headache; GAD-7, Generalized Anxiety Dirorder-7; PHQ-9, Patient Health Questionnare-9; PSS-4, Perceived stress Scale 4; HIT-6, Headache Impact Test-6;ASC-12, Allodynia Symptoms Checklist-12*.

**Table 4:** Comparison of treatment response between definite and probable cluster headache.

	**Definite CH (*N* = 139)**	**Probable CH (*N* = 20)**	***p***
**ACUTE TREATMENT**			
Oxygen	12/14 (85.7%)	1/1 (100.0%)	1.000
NSAIDs	13/55 (23.6%)	5/11 (45.5%)	0.301
Combination analgesics	3/9 (33.3%)	2/4 (50.0%)	1.000
Triptans	83/95 (87.4%)	9/10 (90.0%)	0.476
**PREVENTIVE TREATMENT**			
Steroid	55/62 (88.7%)	8/9 (88.9%)	0.184
Occipital nerve block	13/18 (68.4%)	0/0
Verapamil	69/86 (74.2%)	5/7 (71.4%)	0.743
Lithium	20/30 (66.7%)	2/4(50.0%)	0.471

*CH, cluster headache*.

## Discussion

Three major outcomes were observed in this study. First, the prevalence of probable CH within our patient cohort was 12.6%, and duration of attack was the most common unfulfilled criterion differentiating probable and definite CH. Second, no differences in demographics characteristics, disability, or treatment response were observed between patients with probable and definite CH. Finally, we observed a tendency toward fewer CAS in patients with probable CH, whereas the presence of conjunctival injection and lacrimation was identified as a positive predictor of definite CH.

A previous multicenter, cross-sectional registry study found that a diagnosis of probable primary headache disorder, based on ICHD-3β, was given to 21.3% of first-visit patients due to incomplete or atypical presentations of the headaches. The proportions of probable primary headache disorders differed among the subtypes as follows: migraines (16.1%), tension-type headaches (33%), TACs (40.9%), and other primary headache disorders (14%) (12). Although a difference is noticeable, it is difficult to make a direct comparison because the number of patients included and the method of investigation used differed. In a previous study, the frequency of probable CH, as defined using the ICHD-II guideline, was similar to that in our study, with a frequency of 16.5% (14). In our study, the diagnosis of probable CH was confirmed by re-evaluation at least 2 weeks after the first visit. This research design may help to strengthen the diagnosis of patients with probable CH. The previous longitudinal follow-up study investigated the diagnostic stability of all probable diagnoses using the multicenter headache registry (15). The initial probable headache diagnosis remained unchanged in three-quarters of the patients, with a median follow-up period of 6.5 months. Furthermore, the proportion of consistently diagnosed probable TAC was similar to that of other subtypes of probable primary headache disorders. Based on these results, a diagnosis of probable CH is sufficiently common among CH patients to warrant further study. Such a study would be of importance as little is known about the prevalence and characteristics of probable CH, which is due, in part to the rarity of CH as a primary headache syndrome.

According to two population-based surveys, the most common unmet criterion preventing a definitive migraine diagnosis was headache duration (61.1 and 82.0%, respectively); a separate multicenter study identified the number of attacks (41.9%) and associated symptoms (33%) as the most common unmet symptoms (6, 10, 12). Number of attacks (65.3%) was also the most commonly unmet criterion among cases of probable tension-type headache (12). In terms of unmet criteria, the prevalence of diagnostic stability was lower in subgroups lacking data on time-based criteria, such as number of attacks or total headache period. This lack of data was particularly evident among migraine and tension type headache patients due to the fact that a larger proportion of these patients than of other subgroups of patients progress to a definite diagnosis (15). In our study, the most common unmet criteria were symptom-based, which may be related to atypical presentation. Therefore, the diagnostic stability of probable CH is likely to be confirmed on follow-up. A better understanding of the characteristics of the unmet criteria in probable primary headache disorders would be helpful for differentiating between probable and definitive diagnoses, allowing for better treatment of headache patients (16).

Some of the characteristics of patients with probable CH differed from those with definite CH. Definite CH was characterized by more CAS compared to probable CH. According to the univariate regression analyses, patients with definite CH showed an increased OR for CAS accompanied by conjunctival injection and lacrimation. Of the CAS, lacrimation and conjunctival injection were the most frequent and consistently reported autonomic features in CH (4, 17–22). Several patients diagnosed with probable CH without CAS based on ICHD-II criteria have been included in previous reports, and their clinical profiles have not significantly differed from those with definite CH (14, 23). The only distinction between these two populations was that CH patients without CAS reported less intense attacks relative to patients with CAS, suggesting that CH without CAS may represent a milder form of CH (14). CH attacks without CAS are rare but well known (24, 25), which contradicts the current hypothesis that activation of the parasympathetic pathway is required for the generation of a cluster attack. Such findings suggest that parasympathetic activation is a consequence rather than the cause of trigeminal activation (26).

CH is frequently associated with psychiatric comorbidities. Depression, anxiety, and aggressive behavior are among the most commonly observed psychiatric comorbidities in CH patients (27, 28). These and other symptoms have a substantial impact on the patient's quality of life and, in some cases, can lead to suicidal ideation due to both the frequency and severity of attacks (4). Previous cross-sectional prevalence studies, similar to the one presented here, observed comorbidity rates for depression ranging from 6.3 to 31.0% in episodic CH and from 11.8 to 43.0% in chronic CH (29–33). Similarly, anxiety rates ranged from 15.6 to 23.8% in episodic CH and from 11.8 to 75.7% in chronic CH (29, 30, 32, 33). In our study, the rates of comorbid depression and anxiety were not significantly different between definite and probable CH (41.8 vs. 36.8% in depression and 61.2 vs. 38.9% in anxiety, respectively), with similar mean PHQ-9 and GAD-7 scores, although these rates were higher than those reported in previous studies.

We also assessed headache-related disability and impact on quality of life among patients with probable and definite CH. Previous studies reported disability and reduced quality of life in probable migraine patients relative to controls, and these rates were similar to those of migraine sufferers (5–7, 10). Here, the level of disability and impact on quality of life were comparable in probable and definite CH.

Like migraine headaches, probable migraines are often sub-optimally treated, even in those with access to medical care and prescription drugs (5, 34). Current treatment of probable migraine is based on the assumption that the pathophysiology and treatment response profile of probable migraine are similar to those of migraine (35–38). Cutaneous allodynia, as found for migraine, suggests that central sensitization may also occur in CH (39). Both the extent to which the mechanisms of allodynia are shared across different headache syndromes and the identity of the specific aspects that may be syndrome-specific remain unknown. However, a previous study has suggested that the presence of allodynia not only contributes to our understanding of the pathophysiological mechanism of CH but may also be helpful in determining the treatment and predicting the therapeutic response (40). In our study, there was no significant difference in the presence of or scores for cutaneous allodynia between those with probable CH and those with definite CH. Furthermore, the treatment responses of the two groups did not differ. As probable CH is a CH subtype that responds to specific CH therapy, the same principles of treatment should be applied for both probable and definite CH.

Recently, the new ICHD-3 criteria were published (3). Among the more notable changes, such accompanying symptoms as ipsilateral ear fullness and facial flushing have been excluded for the diagnosis of CH, as the additional diagnostic value of these two symptoms was found to be too low (41). Our study used the ICHD-3β criteria to diagnose CH, which may represent a limitation of this study. To address this potential issue, we compared CH patients diagnosed according to the ICHD-3β criteria with those diagnosed according to the ICHD-3. Although two patients were reclassified by the ICHD-3, there was no significant difference in the clinical profiles of patients diagnosed according to the ICHD-3β and ICHD-3. As this study used a multicenter, clinic-based design, selection bias was inevitable. However, considering the rarity of the condition in the general population, a headache clinic-based study may represent a reasonable setting for evaluation of the clinical features of CH. All patients were followed up for at least 2 weeks, and sufficient data were collected to make a probable diagnosis of CH. This minimum follow-up period may not have been sufficient for assessing the diagnostic consequences of probable CH, and additional long-term follow-up studies will be needed. Additionally, the subgroup analysis of the response to treatment might have been significantly underpowered because few patients actually received each treatment. Finally, recall bias must be considered.

In conclusion, probable CH is a prevalent condition among CH disorders, with a similar disability and impact on quality of life as definite CH. Therefore, both probable and definite CH, which have similar clinical characteristics and impact, deserve similar medical, and therapeutic management.

## Author contributions

JW-P and J-HS conceived the idea for this article. Y-JC, B-KK, P-WC, ML, J-WP, MC, J-YA, B-SK, T-JS, J-HS, KO, K-SL, S-KK, JC, and H-SM contributed the acquisition of the data. K-YP and J-WP performed the data analyses and data interpretations. P-WC, J-WP, MC, J-YA, B-SK, T-JS, J-HS, KO, K-SL, S-KK, JC, H-SM, and C-SC contributed with inputs to this article. J-HS and J-WP drafted the work and paper. B-KK, P-WC, ML, J-WP, MC, J-YA, B-SK, T-JS, J-HS, KO, K-SL, S-KK, K-YP, JC, H-SM, and C-SC revised the paper for important intellectual content. All authors reviewed and approved the final manuscript. All authors agreed to be accountable aspects of the work in ensuring that questions related to the accuracy or integrity of any part of the work are appropriately investigated and resolved.

### Conflict of interest statement

MC was involved as a site investigator for a multicenter trial sponsored by Eli Lilly, worked an advisory member for Teva, and received lecture honoraria from Allergan Korea and Yuyu Pharmaceutical Company. S-JC was involved as a site investigator of multicenter trial sponsored by Otsuka Korea, Eli Lilly and Company, Korea BMS, and Parexel Korea Co., Ltd., and received research support from Hallym University Research Fund 2016 and Myungin Research Fund 2016. The remaining authors declare that the research was conducted in the absence of any commercial or financial relationships that could be construed as a potential conflict of interest.

## Abbreviations

CH: Cluster headache
CAS: Cranial Autonomic Symptom
ICHD: International Classification of Headache Disorder
TAC: Trigeminal Autonomic Cephalalgia
ICHD-3β: International Classification of Headache Disorder, 3rd Edition, beta version
HIT-6: Headache Impact Test-6
PHQ-9: Patient Health Questionnare-9
GAD-7: Generalized Anxiety Dirorder-7
PSS-4: Perceived Stress Scale-4.
